# Dermatomyositis with anti-TIF-1γ antibodies as a presenting symptom of underlying triple-negative breast cancer: a case report

**DOI:** 10.1186/s12885-016-2715-1

**Published:** 2016-08-25

**Authors:** Ondřej Kubeček, Tomáš Soukup, Adam Paulík, Jindřich Kopecký

**Affiliations:** 1 Department of Oncology and Radiotherapy, Charles University in Prague, Faculty of Medicine and University Hospital in Hradec Králové, Sokolská 581, 500 05 Hradec Králové, Czech Republic; 2 2nd Department of Internal Medicine – Gastroenterology, Charles University in Prague, Faculty of Medicine and University Hospital in Hradec Králové, Sokolská 581, 500 05 Hradec Králové, Czech Republic

**Keywords:** Autoantibodies, Breast cancer, Dermatomyositis, Paraneoplastic, Case report

## Abstract

**Background:**

Dermatomyositis is an autoimmune myopathy characterized by proximal muscle weakness, muscle inflammation, and typical skin findings. It is a rare disease with an incidence of ~1/100 000. About 15–30 % of adult-onset cases are caused by underlying malignancy and dermatomyositis can be the first symptom of undiagnosed cancer, mainly in the case of anti-transcription intermediary factor 1γ (anti-TIF-1γ) antibodies presence. TIF-1γ is a transcriptional cofactor which is implicated in TGFβ signaling pathway that controls cell proliferation, differentiation, apoptosis, and tumorigenesis. Its expression was shown to be associated with younger age, higher tumor grade, more estrogen receptor negativity, tumors larger than 2 cm, and tendency towards poor outcome in early breast cancer. No association between anti-TIF-1γ antibodies and prognosis has been proposed yet.

**Case presentation:**

We report a case of a 43-year-old premenopausal woman presenting with the symptoms of systemic rheumatic disease, the most prominent being a typical skin rash and muscle pain. After a series of investigations, the patient was diagnosed with anti-TIF-1γ positive dermatomyositis and concurrent triple-negative breast cancer (cT1c N3c M0) as an underlying cause. Immediate intravenous corticosteroid therapy relieved the symptoms and enabled anticancer therapy to be commenced. Considering the tumor stage, neoadjuvant therapy with 4 courses of AC (Doxorubicin/Cyclophosphamide) followed by 4 courses of Paclitaxel/Carboplatin was administered. However, no tumor regression was documented and radiotherapy was chosen as the definitive treatment.

**Conclusion:**

Early detection of anti-TIF-1γ autoantibodies can contribute to a rapid diagnosis of tumor-associated dermatomyositis and enable immediate anticancer treatment. We demonstrate the emerging role of anti-TIF-1γ antibodies in the diagnostics of tumor-associated dermatomyositis. Furthermore, we propose a potential role of anti-TIF-1γ antibodies as a prognostic marker in early breast cancer patients.

## Background

Dermatomyositis (DM) together with polymyositis and inclusion body myositis belong to a group of acquired skeletal muscle diseases known as idiopathic inflammatory myopathies [1]. DM is considered an autoimmune disease. It may present with variously expressed clinical signs, the most prominent being characteristic rash and muscle weakness. The skin manifestations include heliotrope rash on the upper eye lids, erythematous rash localized on the face, neck, anterior chest, back, and large joints [2]. Gottron rash refers to violaceous rash or papules localized typically in metacarpophalangeal, and proximal or distal interphalangeal joints. Together with heliotrope rash, it is considered a specific cutaneous feature of the disease. Proximal muscle weakness usually develops slowly over weeks or months [3]. DM is associated with presence of specific autoantibodies which are usually divided into myositis specific autoantibodies (including anti-Mi-2, anti-CADM-140, anti-SAE, anti-p155/140 (anti-TIF-1γ), anti-MJ, anti-t-RNA synthetase, and anti-PMS1 antibodies) and myositis associated autoantibodies (including anti-Ro/SSA, anti-U1RNP, anti PM/Scl, and Anti-Ku antibodies) [4]. Recently, a remarkable association between several antibodies and specific clinical presentations have been found [5]. The majority of cases are idiopathic. However, in ~15–30 % of adult-onset cases, DM is associated with malignancy. Cancer may occur before, at the same time, or following the diagnosis of DM. DM that develops as a consequence of the tumor presence in the body is classified as paraneoplastic. The mechanism how malignancy induces DM is not clear yet. However, several possible mechanisms have been proposed. It has been demonstrated that some tumors, including breast adenocarcinoma, express high levels of myositis autoantigens [6]. These antigens can also be found in regenerating myoblasts in affected muscles from myositis patients. It is therefore possible that immune response directed against cancer cells cross-reacts with regenerating muscle cells and could therefore be responsible for the pathogenesis of DM [6]. The most common tumor types associated with DM include gynecological tumors (mainly ovarian cancer), lung, pancreatic, gastric, colorectal cancer, and non-Hodgkin’s lymphoma [7]. The most common tumor sites in men are lung, prostate, and stomach. This contrasts with DM in women, which is most frequently associated with tumors of breast, ovary, and uterus [8]. The spectrum of tumor types varies greatly across different regions. DM in Asian populations is, for example, more frequently associated with a nasopharyngeal cancer [9]. The first case of DM associated with breast cancer was reported in 1916 [10]. According to published population-based studies, breast cancer is diagnosed in ~10–20 % of malignancy-associated DM cases [8].

Triple-negative cancer is an intrinsic subtype of breast cancer. It is defined by the absence of estrogen receptor (ER), progesterone receptor (PgR), and human epidermal growth factor receptor 2 (HER2) overexpression and/or gene amplification [11]. It accounts for 15–20 % of newly diagnosed breast cancer cases [12]. Typical features include younger age at diagnosis, poorer prognosis, and association with BRCA 1/2 mutations [12]. No data regarding the incidence of anti-TIF-1γ autoantibodies in triple-negative breast cancer has been published so far.

## Case presentation

A forty-three-year-old premenopausal caucasian woman with no relevant medical history presented with intermittent fever, fatigue, myalgia, dysphagia, and erythematous rash lasting over 1 month. The nonpruritic macular rash was initially localized on the back of patient’s hands and was followed by eruption of erythema over patient’s face after 1 week. The onset of pruritic macular rash on lateral sites of patient’s thighs and upper part of back followed. The patient presented first to her dermatologist who treated her with topical steroids for 2 weeks. However, no clinical effect was observed. Considering the skin manifestation and other symptoms suspicious of a systemic rheumatic disease, the patient was referred to a rheumatologist. She was later admitted to a hospital to establish a diagnosis and commence treatment.

**Fig. 1:**
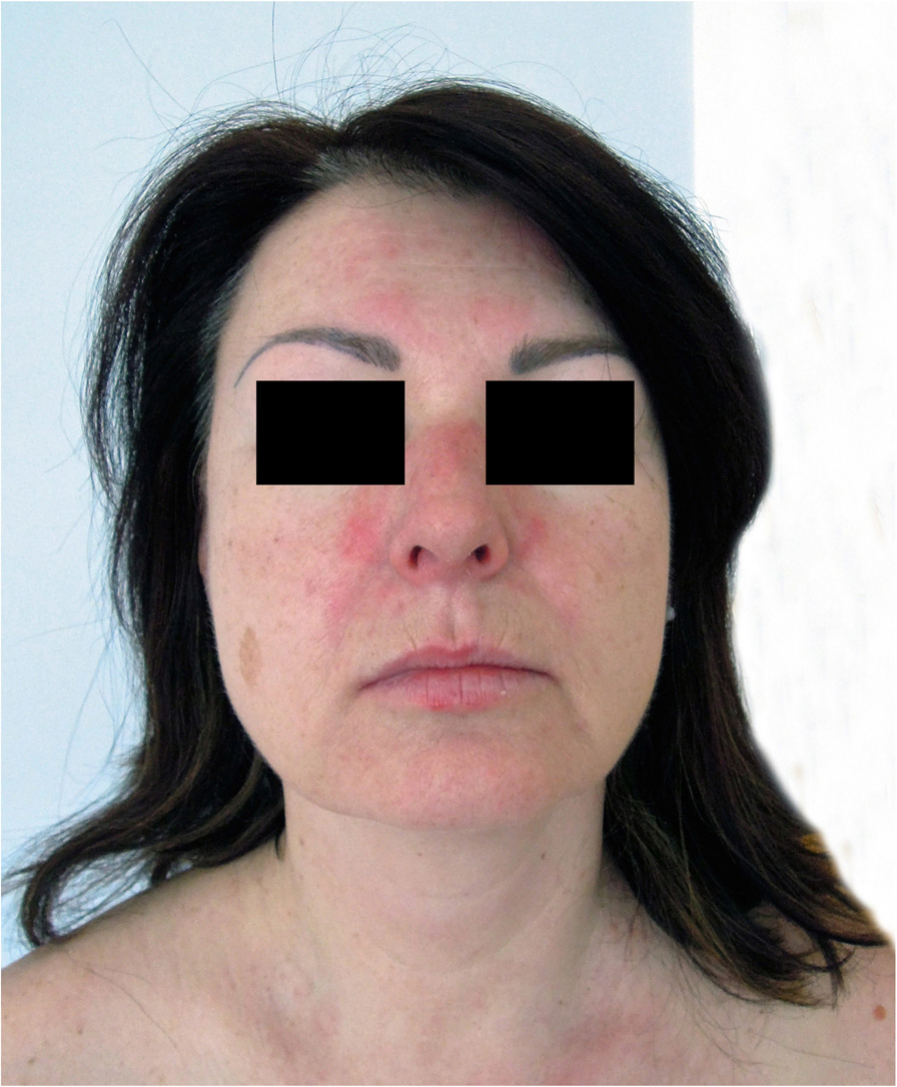
Erythematous rash localized over the cheeks and nasal bridge omitting the nasolabial sulcus resembling a typical malar rash in lupus erythematosus

**Fig. 2:**
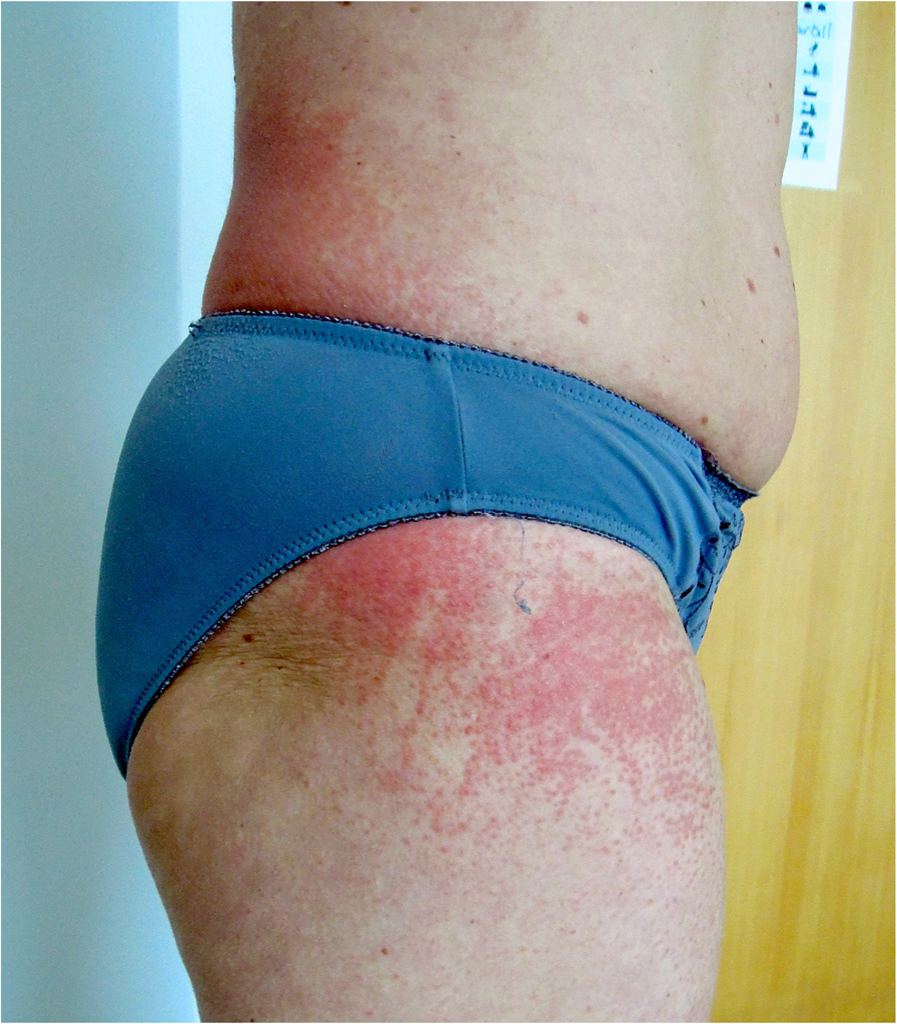
Maculopapular rash on lateral sites of patient’s thighs

**Fig. 3:**
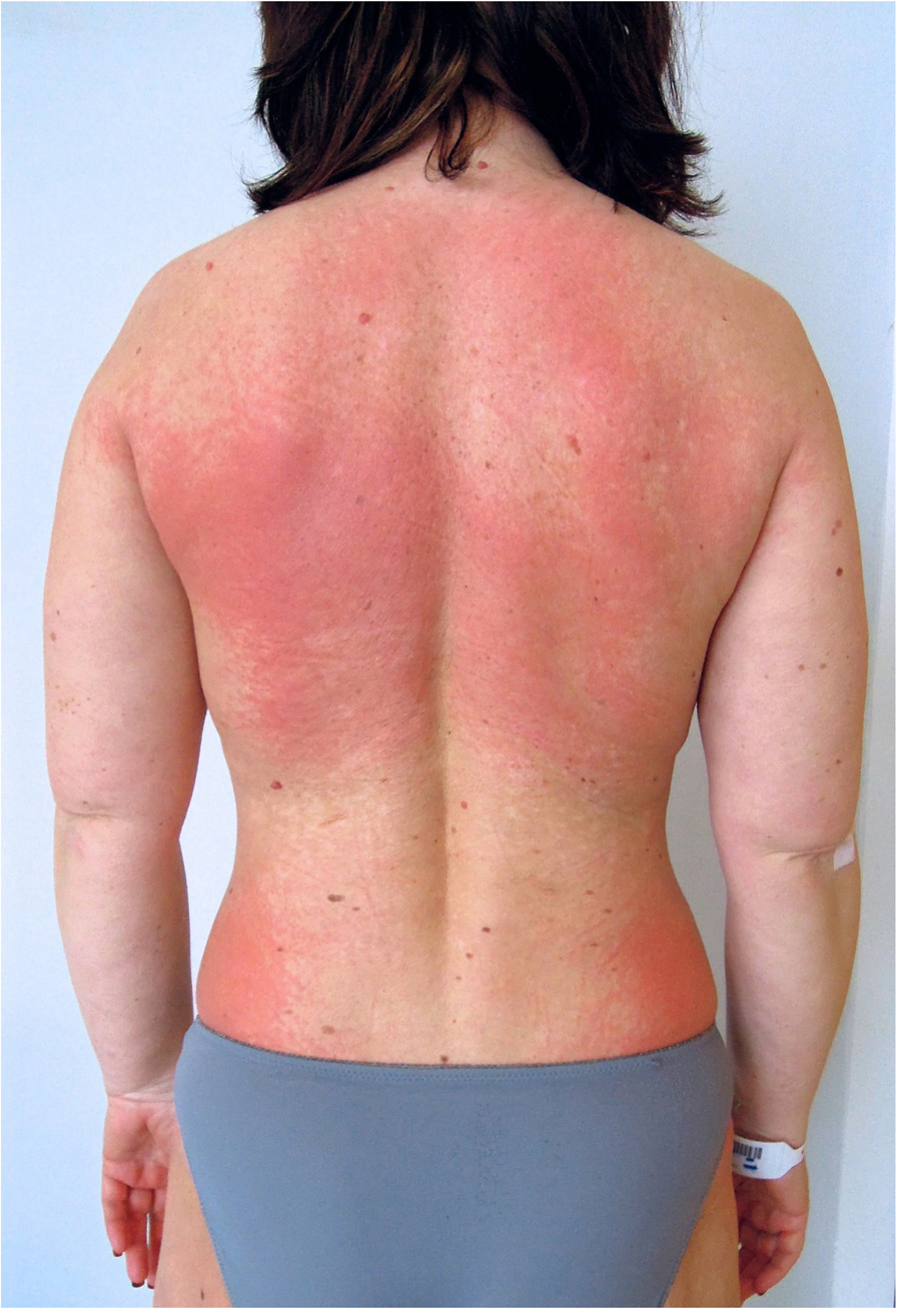
Maculopapular rash on patient’s back

**Fig. 4:**
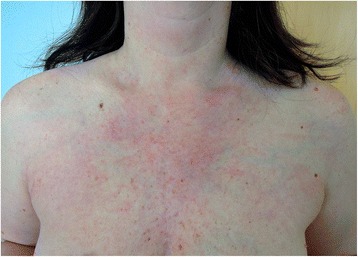
V-sign (macular exanthema on the front site of patient’s chest)

**Fig. 5:**
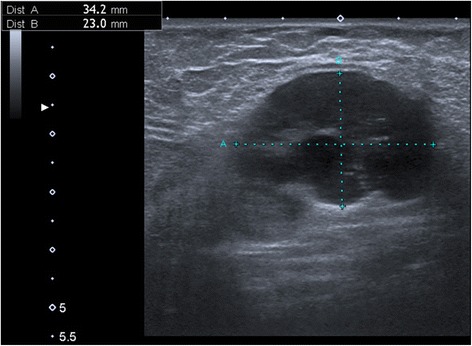
Ultrasound image of enlarged lymph node in the left axilla

The skin examination revealed a slight periorbital edema (Heliotrope rash) and erythematous rash localized over her cheeks and nasal bridge omitting the nasolabial sulcus. It was therefore resembling a typical malar rash in lupus erythematosus (Fig. 1). Erythematous hyperpigmented papules were found over the proximal and distal interphalangeal joints (Gottron’s sign). The pruritic maculopapular rash was also present on lateral sites of patient’s thighs and back (Figs. 2 and 3). V-sign, a typical distribution of macular exanthema on the front site of patient’s chest, was found (Fig. 4). The physical examination also revealed enlarged lymph nodes in her left axilla and supraclavicular region, which was further confirmed by ultrasound (Fig. 5), and painful swelling and enlargement of the whole left mammary gland.

**Fig. 6:**
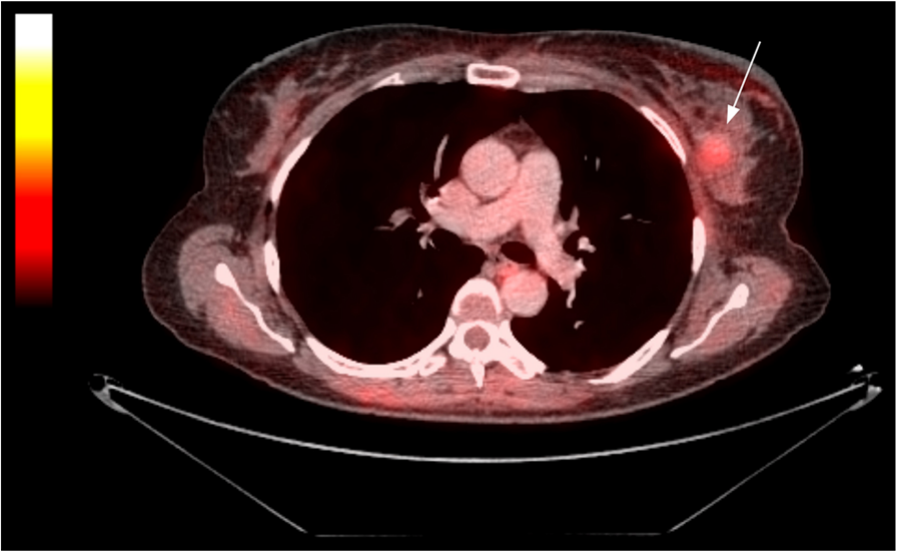
PET/CT showing FDG-avid lesion 19 × 14 mm in the left mammary gland

**Fig. 7:**
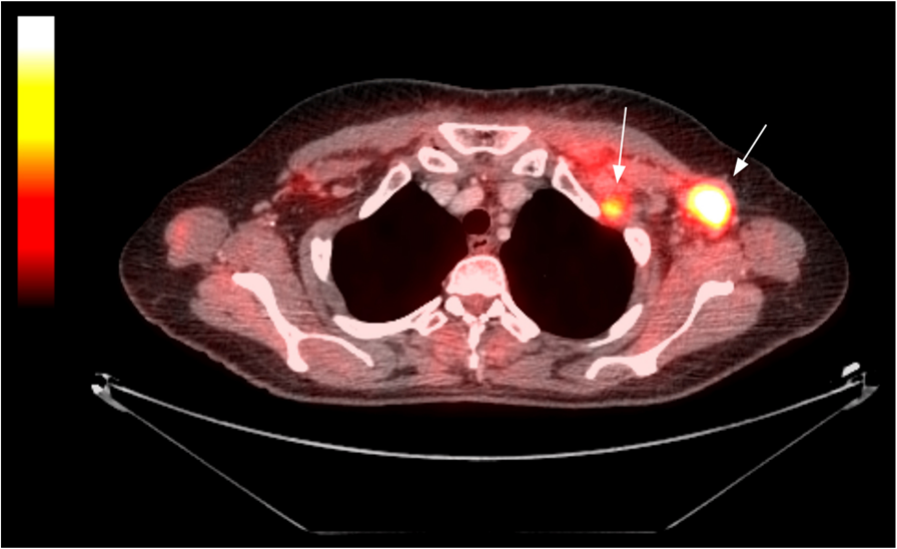
PET/CT showing multiple FDG-avid lymph nodes in the left axilla and under the pectoralis major muscle

Blood investigations revealed raised erythrocyte sedimentation rate (ESR, 34 mm/h). C-reactive protein (CRP) was slightly elevated (12.4 mg/L). Serum muscle enzyme concentrations were also elevated - alanine aminotransferase (0.71 μkat/L), aspartate aminotransferase (1.38 μkat/L), creatine kinase (CK, 26.24 μkat/L), and myoglobin (267 μg/L). Electromyography revealed no pathological findings. Skin biopsy from the affected area was performed with negative lupus band test (direct immunofluorescence test for lupus erythematosus). The whole panel of autoantibodies was examined using immunoblot technique (Euroimmun, Euroline Autoimmune Inflammatory Myopathies 16 Ag) with only anti-TIF-1γ antibodies being positive. The probability of DM was evaluated > 90 % using the IMCCP (The International Myositis Classification Criteria Project) criteria [13]. Muscle biopsy is not mandatory in the presence of typical skin manifestations according to these criteria. Considering the high probability of DM, further supported by the presence of anti-TIF-1γ, a muscle biopsy was omitted. Because of the patient’s complains of dysphagia gastro-duodenoscopy was performed to exclude the upper gastrointestinal tract involvement. The finding was consistent with erosive antral gastritis with negative rapid urease test (rapid diagnostic test for *Helicobacter pylori* infection). Series of investigations were performed to exclude an extra-muscular involvement - ophthalmologic, otorhinolaryngologic, neurologic, cardiac and renal - all with negative findings. Tumor markers including CEA, CA 15–3, CA 125, and CA 19–9 were negative. Mammography was performed to exclude breast cancer as an underlying condition. The result, however, was equivocal (sporadic microcalcifications without any obvious tumor mass). Neither ultrasound of the abdomen nor chest x-ray showed any abnormality. Since the conventional investigations were not conclusive, a whole body PET/CT scan was performed to exclude malignancy. The scan showed FDG-avid lesion 19 × 14 mm in the left mammary gland, and multiple FDG-avid lymph nodes in the left axilla and under the pectoralis major muscle (Figs. 6 and 7).

**Fig. 8:**
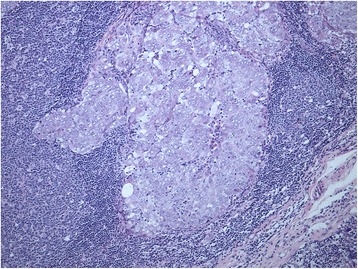
Light microscope image of lymph node infiltrated with metastasis of poorly differentiated adenocarcinoma (Haematoxylin and Eosin stain)

Direct core cut biopsy was not feasible since there was no evident mass within the breast according to mammography. Extirpation of the left supraclavicular lymph node was therefore performed to obtain the histopathological specimen and confirm the diagnosis. Metastasis of poorly differentiated adenocarcinoma was reported (Fig. 8). Immunohistochemical assessment of steroid hormone status was performed. The expression of the estrogen and progesteron receptors, and HER-2/neu protein were all negative. Additional in situ hybridization (ISH) found no HER-2/neu amplification, thus confirming the diagnosis of the triple-negative breast cancer. The disease was staged as cT1c N3c M0. Genetic testing to exclude BRCA 1/2 mutation was advised, considering the patient’s age and histological type of breast cancer (triple-negative).

The treatment of DM was initiated with intravenous pulse corticosteroid therapy (methylprednisolone 5 × 1000 mg alternate days), followed by oral corticosteroids. Initial daily dose of 32 mg of methylprednisolone was reduced by 8 mg every week to the maintenance dose of 8 mg/day. The muscle strength tended to deteriorate during the deescalation phase. This prompted the contemporary discontinuation of dose reduction, until muscle weakness improved (CK 1.03 μkat/L, myoglobin 25.5 μg/L). The symptoms of dysphagia and skin manifestation were promptly managed, enabling the anti-cancer therapy to be commenced. Considering both disease burden and histologic features, neoadjuvant chemotherapy was decided to be the option. The regimen containing 4 courses of doxorubicin 60 mg/m^2^ + cyclophosphamide 600 mg/m^2^ q3w followed by 4 courses paclitaxel 175 mg/m^2^ + carboplatin AUC 5 q3w was chosen. The patient continued with a maintenance dose of corticosteroids during the chemotherapy, which was well tolerated. However, the investigations following the last dose of chemotherapy (mammography, ultrasound of the breast and regional lymph nodes), did not show any signs of tumor regression. Surgery was still not feasible because of the supraclavicular and axillary lymph node persistence. Radiotherapy was therefore decided to be the option by the multidisciplinary board (including oncologist, breast surgeon and radiologist) in order to achieve local control. Primary chemoresistance of the tumor was an additional reason to support this approach.

## Discussion

Cancer should always be considered as an underlaying cause of DM. Several risk factors have been proposed to be related with increased risk of malignancy in DM patients. An extensive meta-analysis of clinical trials found older age, male sex, dysphagia, cutaneous necrosis, cutaneous vasculitis, rapid onset (<4 weeks), elevated CK, higher ESR, and higher CRP as factors to be associated with higher risk [14]. Considering the high incidence of cancer in DM patients, it is the author’s opinion that every patient with DM should be thoroughly investigated to exclude the malignancy with an extra effort in patients bearing one or more of above named risk factors.

The diagnosis of DM is traditionally based on five criteria published by Bohan and Peter in 1975: 1) Symmetric proximal muscle weakness, 2) Muscle biopsy evidence of myositis, 3) Increase in serum skeletal muscle enzymes, 4) Characteristic electromyographic patterns, and 5) Typical rash of dermatomyositis. Two of these criteria were present together with typical skin manifestation which made the diagnosis of DM probable in presented case [15]. However, these criteria seem to be inadequate in several aspect (e.g. including patients with some forms of muscle dystrophy). Novel criteria suitable to be used within clinical trials have therefore been proposed recently [13]. Using the IMCCP criteria (The International Myositis Classification Criteria Project) in our patient, the probability of DM was evaluated > 90 %.

Anti-TIF-1γ antibodies were positive while anti-NXP-2 (anti-nuclear matrix protein NXP-2) antibodies were negative in presented patient. It has been shown that the presence of anti-TIF-1γ and anti-NXP-2 antibodies is frequent in DM patients [5]. These autoantibodies are present in most patients with cancer-associated DM (found in 83 % cases - 31 % anti-NXP-2 and 52 % anti-TIF-γ), which could make them a useful tool to identify patients with malignancy alongside the DM [5]. Furthermore, it was observed that these antibodies are almost exclusively non-overlapping and that the vast majority of individuals with positivity of either antibody is negative in other DM-specific or myositis-specific antibodies as observed in our patient [5]. Thus, the involvement of these antibodies in the primary diagnostics of DM could identify patients with otherwise negative autoantibodies.

TIF-1γ is a transcriptional cofactor which is implicated in TGFβ signaling pathway that controls cell proliferation, differentiation, apoptosis and tumorigenesis [16]. Results of a study evaluating the role of TIF-1γ and its interaction with TGFβ1/SMAD4 signaling pathway as a prognostic factor in operable breast cancer have been published recently [17]. TIF-1γ expression was shown to be associated with younger age, higher tumor grade, more estrogen receptor (ER) negativity, and tumors larger than 2 cm [17]. Furthermore, TIF1γ expression showed tendency towards poor outcome. The subgroup of patients expressing both TGFβ1 and TIF1γ showed the poorest outcome in the studied population [17]. We propose that if the correlation of serum anti-TIF-1γ antibodies and TIF-1γ expression in the tumor was found, the anti-TIF-1γ antibodies might serve as a potential prognostic marker in early breast cancer patients.

DM is considered a potentially treatable disease. Systemic corticosteroids remain a mainstay in therapy. Oral prednisone at initial dose of 0.5–1 mg/kg/day should be given as initial therapy, followed by a slow progressive dose reduction not earlier than 6 weeks after the myositis has become inactive (clinically and enzymatically) [18]. In severe cases, intravenous methylprednisolone is the treatment option [19]. Some patients, however, do not respond to corticosteroids or develop serious side effects. In such cases, introduction of immunosuppressive agents is recommended. The most widely used drugs include methotrexate, azathioprine, cyclophosphamide or cyclosporin A [3]. The surgical removal of the tumor or anti-cancer treatment may itself result in disappearance or reduction of the paraneoplastic symptoms [20]. Methylprednisolone pulse therapy seems to relieve dysphagia in DM patients [21]. This finding together with urgency to achieve a rapid response was the reason to choose the initial pulse corticotherapy. The symptoms of the disease were managed soon after making the diagnosis and the patient was ready to begin the anti-cancer treatment without undue delay.

## Conclusions

DM can be the first symptom of previously undiagnosed malignancy. When confirming the diagnosis of DM, malignancy should always be excluded. The anti-TIF-1γ and anti-NXP-2 antibodies may play an essential role in rapid diagnosis of malignancy associated DM, especially in patients bearing several risk factors known to be associated with malignancy. Considering the fact that individuals with positivity of either of these antibodies are very frequently negative in other DM-specific and myositis-specific antibodies, their assessment within primary diagnostics might be beneficial to identify patients with otherwise negative antibodies.

Proper management of DM, including the use of intravenous corticosteroids, is essential to enable early cancer treatment. Maintenance corticosteroid therapy does not interfere with chemotherapy administration and can provide control of the rheumatic disease during cancer treatment.

Furthermore, we propose that anti-TIF-1γ antibodies might serve as a prognostic marker of worse clinical outcome in early breast cancer provided correlation between serum anti-TIF-1γ antibodies and TIF-1γ expression in the tumor is found.

## Abbreviations

AUC: Area under the curve
CEA: Carcinoembryonic antigen
CK: Creatine kinase
CRP: C-reactive protein
DM: Dermatomyositis
ER: Estrogen receptor
ESR: Erythrocyte sedimentation rate
FDG: Fluorodeoxyglucose
HER2: Human epidermal growth factor receptor 2
IMCCP: The International Myositis Classification Criteria Project
ISH: In situ hybridization
pCR: Pathologic complete response
PET/CT: Positron emission tomography-computed tomography
PgR: Progesteron receptor
TGFβ: Transforming growth factor β
TIF-1γ: Transcription intermediary factor 1γ

## References

[1] Dalakas MC (1991). Polymyositis, dermatomyositis and inclusion-body myositis. N Engl J Med.

[2] Dalakas MC, Hohlfeld R (2003). Polymyositis and dermatomyositis. Lancet.

[3] Callen JP, Wortmann RL (2006). Dermatomyositis. Clin Dermatol.

[4] Iaccarino L, Ghirardello A, Bettio S, Zen M, Gatto M, Punzi L, Doria A (2014). The clinical features, diagnosis and classification of dermatomyositis. J Autoimmun.

[5] Fiorentino DF, Chung LS, Christopher-Stine L, Zaba L, Li S, Mammen AL, Rosen A, Casciola-Rosen L (2013). Most patients with cancer-associated dermatomyositis have antibodies to nuclear matrix protein NXP-2 or transcription intermediary factor 1gamma. Arthritis Rheum.

[6] Casciola-Rosen L, Nagaraju K, Plotz P, Wang K, Levine S, Gabrielson E, Corse A, Rosen A (2005). Enhanced autoantigen expression in regenerating muscle cells in idiopathic inflammatory myopathy. J Exp Med.

[7] Hill CL, Zhang Y, Sigurgeirsson B, Pukkala E, Mellemkjaer L, Airio A, Evans SR, Felson DT (2001). Frequency of specific cancer types in dermatomyositis and polymyositis: a population-based study. Lancet.

[8] Barnes BE, Mawr B (1976). Dermatomyositis and malignancy. A review of the literature. Ann Intern Med.

[9] Peng JC, Sheen TS, Hsu MM (1995). Nasopharyngeal carcinoma with dermatomyositis. Analysis of 12 cases. Arch Otolaryngol Head Neck Surg.

[10] Kankeleit H (1916). Über primäre nichteitrige Polymyositis. Dtsch Arch Klin Med.

[11] Reis-Filho JS, Tutt AN (2008). Triple negative tumours: a critical review. Histopathology.

[12] Bauer KR, Brown M, Cress RD, Parise CA, Caggiano V (2007). Descriptive analysis of estrogen receptor (ER)-negative, progesterone receptor (PR)-negative, and HER2-negative invasive breast cancer, the so-called triple-negative phenotype: a population-based study from the California cancer Registry. Cancer.

[13] Pilkington C (2014). Tjärnlund A, Bottai M, Werth V, Visser M, Alfredsson L, Amato A, Barohn RJ, Liang M, Singh J et al.: Progress report on development of classification criteria for adult and juvenile idiopathic inflammatory myopathies. Pediatric. Rheumatology.

[14] Lu X, Yang H, Shu X, Chen F, Zhang Y, Zhang S, Peng Q, Tian X, Wang G (2014). Factors predicting malignancy in patients with polymyositis and dermatomyostis: a systematic review and meta-analysis. PLoS One.

[15] Bohan A, Peter JB (1975). Polymyositis and dermatomyositis (first of two parts). N Engl J Med.

[16] Wakefield LM, Piek E, Bottinger EP (2001). TGF-beta signaling in mammary gland development and tumorigenesis. J Mammary Gland Biol Neoplasia.

[17] Kassem L, Deygas M, Fattet L, Lopez J, Goulvent T, Lavergne E, Chabaud S, Carrabin N, Chopin N, Bachelot T (2015). TIF1gamma interferes with TGFbeta1/SMAD4 signaling to promote poor outcome in operable breast cancer patients. BMC Cancer.

[18] Oddis CV (1994). Therapy of inflammatory myopathy. Rheum Dis Clin North Am.

[19] Marie I, Mouthon L (2011). Therapy of polymyositis and dermatomyositis. Autoimmun Rev.

[20] Racanelli V, Prete M, Minoia C, Favoino E, Perosa F (2008). Rheumatic disorders as paraneoplastic syndromes. Autoimmun Rev.

[21] Hrncir Z (1992). Favorable effect of methylprednisolone pulse therapy in dysphagia and primary idiopathic polymyositis/dermatomyositis. Cas Lek Cesk.

